# Genetic diversity of Newcastle disease virus in Pakistan: a countrywide perspective

**DOI:** 10.1186/1743-422X-10-170

**Published:** 2013-05-30

**Authors:** Muhammad Zubair Shabbir, Siamak Zohari, Tahir Yaqub, Jawad Nazir, Muhammad Abu Bakr Shabbir, Nadia Mukhtar, Muhammad Shafee, Muhammad Sajid, Muhammad Anees, Muhammad Abbas, Muhammad Tanveer Khan, Asad Amanat Ali, Aamir Ghafoor, Abdul Ahad, Aijaz Ali Channa, Aftab Ahmad Anjum, Nazeer Hussain, Arfan Ahmad, Mohsan Ullah Goraya, Zahid Iqbal, Sohail Ahmad Khan, Hassan bin Aslam, Kiran Zehra, Muhammad Umer Sohail, Waseem Yaqub, Nisar Ahmad, Mikael Berg, Muhammad Munir

**Affiliations:** 1 Quality Operations Laboratory, University of Veterinary and Animal Sciences, Lahore, Pakistan; 2 National Veterinary Institute, Uppsala, Sweden; 3 Department of Microbiology, University of Veterinary and Animal Sciences, Lahore, Pakistan; 4 University of Baluchistan, Quetta, Pakistan; 5 Veterinary Research and Disease Investigation Center, Abbottabad, Pakistan; 6 Livestock and Dairy Development Department, Punjab, Pakistan; 7 Veterinary Research Institute, Lahore, Pakistan; 8 Department of Biology, University of Bergen, Bergen, Norway; 9 Poultry Research Institute, Rawalpindi, Pakistan; 10 University Diagnostic Laboratory, University of Veterinary and Animal Sciences, Lahore, Pakistan; 11 Department of Microbiology, Chittagong Veterinary and Animal Sciences University, Chittagong, Bangladesh; 12 Department of Theriogenology, University of Veterinary and Animal Sciences, Lahore, Pakistan; 13 Sindh Poultry Vaccine Center, Karachi, Pakistan; 14 Swedish University of Agricultural Sciences, Uppsala, Sweden; 15 Department of Geography, Government College University, Lahore, Pakistan; 16 Government Collage University Faisalabad, Faisalabad, Pakistan; 17 Department of Parasitology, University of Veterinary and Animal Sciences, Lahore, Pakistan; 18 The Pirbright Institute, Compton Laboratory, Compton, Berkshire RG20 7NN, United Kingdom

**Keywords:** Lineages, Newcastle disease virus, Pakistan, Phylogenetic analysis, Poultry

## Abstract

**Background:**

Newcastle disease (ND) is one of the most deadly diseases of poultry around the globe. The disease is endemic in Pakistan and recurrent outbreaks are being reported regularly in wild captive, rural and commercial poultry flocks. Though, efforts have been made to characterize the causative agent in some of parts of the country, the genetic nature of strains circulating throughout Pakistan is currently lacking.

**Material and methods:**

To ascertain the genetics of NDV, 452 blood samples were collected from 113 flocks, originating from all the provinces of Pakistan, showing high mortality (30–80%). The samples represented domesticated poultry (broiler, layer and rural) as well as wild captive birds (pigeons, turkeys, pheasants and peacock). Samples were screened with real-time PCR for both matrix and fusion genes (1792 bp), positive samples were subjected to amplification of full fusion gene and subsequent sequencing and phylogenetic analysis.

**Results:**

The deduced amino acid sequence of the fusion protein cleavage site indicated the presence of motif (^112^RK/RQRR↓F^117^) typical for velogenic strains of NDV. Phylogenetic analysis of hypervariable region of the fusion gene indicated that all the isolates belong to lineage 5 of NDV except isolates collected from Khyber Pakhtunkhwa (KPK) province. A higher resolution of the phylogenetic analysis of lineage 5 showed the distribution of Pakistani NDV strains to 5b. However, the isolates from KPK belonged to lineage 4c; the first report of such lineage from this province.

**Conclusions:**

Taken together, data indicated the prevalence of multiple lineages of NDV in different poultry population including wild captive birds. Such understanding is crucial to underpin the nature of circulating strains of NDV, their potential for interspecies transmission and disease diagnosis and control strategies.

## Introduction

Newcastle disease is caused by avian paramyxovirus serotype-1 (APMV-1), which is also known as Newcastle disease virus (NDV). It is a highly contagious viral disease that affects domesticated and wild bird species throughout the world [1-4]. The disease is considered enzootic in Pakistan and represents major threat to the economy of the country. However, disease severity is variable in different host species and in different geographical locations.

NDV is classified in the genus *Avulavirus* within subfamily *Paramyxovirinae*, family *Paramyxoviridae* and order *Mononegavirales*[1]. This enveloped virus has a negative-sense, non-segmented, single stranded RNA genome of 15186, 15192 or 15198 nucleotides in length [1,5,6]. The genome encodes six structural and two non-structural proteins [6]. Based on the fusion (F) gene sequence, NDV strains are classified into lineages or genotypes; however, the discrepancies between the two classification systems are nominal [5,7-10]. Clinical manifestation or severity of the ND depends largely upon the isolates involved in disease outbreak [1,2]. Based upon pathogenicity, these strains are commonly categorized into velogenic, mesogenic and lentogenic types [4]. The varying level of pathogenicity is attributed to amino acid sequence motif present in the protease cleavage site of the precursor F protein [11]. The amino acid sequence in more virulent, velogenic and mesogenic strains is ^112^R/K-R-Q-R/K-R↓F^117^. This sequence is cleavable by a variety of cellular proteases in various organs, resulting in wider systemic infection in respiratory system, gastro-intestinal tract and nervous system. The sequence in less virulent, lentogenic strains of NDV is ^112^G/E-K/R-Q-G/E-R↓L^117^. This sequence is cleavable only by trypsin like proteases, hence limiting the infection only to respiratory system and gastro-intestinal tract [1,4,6].

Pakistan has an agriculture based economy with livestock and poultry as an integral part of it. Nearly every family in the rural areas and every 5th family in the urban areas is associated with poultry in one way or the other [12]. The poultry has emerged as the second largest industry in Pakistan with an annual increase of 4%, supplying eggs and meat as the protein sources [13]. Since the development of an organized poultry sector in Pakistan, the Newcastle disease has caused havocs to the poultry industry several times. Appropriate vaccination and subsequent effective immune response is known to be the only measure to avoid the disease outbreaks, but this approach cannot be extended logistically to all domesticated and wild birds. Large number of disease outbreaks was recorded during 2010–12 in Pakistan.

Reports about the disease in Pakistan have largely been focused to Punjab province, reporting existence of velogenic NDV strains [9,11,14-17]. However, a systematic investigation of genetic nature of NDV strains in clinically diseased flocks throughout Pakistan has been lacking so far. Failure of previously effective live vaccines in protecting the birds from current field isolates stimulated us to investigate the genetic relationship between the past and current viral isolates. This is crucial due to the fact that disease continue to appear in both vaccinated flocks as well as unvaccinated commercial, wild captive and backyard poultry birds. This is the first report that encompasses the molecular characterization of prevailing NDV strains from across Pakistan.

## Materials and methods

### History and collection of samples

**Figure 1:**
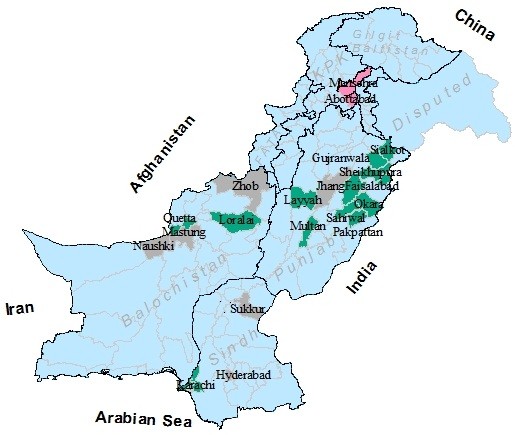
Sampling sites and geographical distribution of Newcastle disease virus lineages in Pakistan.

To ascertain the genetic nature of circulating NDV strains in Pakistan, blood samples were collected during an emerging wave of the disease from February to July 2012. A total of 113 flocks originating from various districts in the province of Punjab, Khyber Pakhtunkhwa, Sindh and Baluchistan were examined (Table 1, Figure 1). The under-study flocks consist of commercial (n = 60), rural (n = 42) and domesticated birds including pigeon, turkeys and peacocks (n = 11). From each diseased flock, whole blood (3–5 mL) was collected aseptically from brachial vein of four diseased birds randomly in an anticoagulant added vaccutainer (Venoject^(R)^, Belgium). A brief history regarding age at infection, clinical symptoms, mortalities and course of infection was recorded.

**Table 1:** Detail of samples collected from districts and diagnostic efficacy of real-time PCR

**Province**	**District**	**Type of birds**											
		^**1**^**Commercial**				^**2**^**Rural**				^**3**^**Wild captive**			
		**Flock (n)**	**Blood samples (n)**	^**4**^**Real time PCR**		**Flock (n)**	**Blood samples (n)**	**Real Time PCR**		**Flock (n)**	**Blood samples (n)**	**Real time PCR**	
				**M gene**	**F gene**			**M gene**	**F gene**			**M gene**	**F gene**
Baluchistan	Quetta	09	36	+	+	03	12	+	+	0.0	0.0	-	-
	Noshki	0.0	0.0	-	-	04	16	-	-	0.0	0.0	-	-
	Zhob	0.0	0.0	-	-	01	04	-	-	0.0	0.0	-	-
	Mastoung	0.0	0.0	-	-	02	08	-	-	0.0	0.0	-	-
	Loralai	09	03	+	+	05	20	+	+	0.0	0.0	-	-
KPK	Abbottabad	11	44	+	+	0.0	0.0	-	-	0.0	0.0	-	-
	Mansehra	10	40	+	+	0.0	0.0	-	-	0.0	0.0	-	-
Sindh	Karachi	06	24	+	+	02	08	+	+	0.0	0.0	-	-
	Hyderabad	03	12	-	-	03	12	-	-	0.0	0.0	-	-
	Sukkur	01	04	-	-	02	08	-	-	0.0	0.0	-	-
Punjab	Okara	02	08	+	+	01	04	+	+	04 (turkey and pigeon	16	+	+
	Faisalabad	10.0	0.3	+	+	07	28	+	+	02 (pigeon)	08	+	+
	Layyah	12	04	+	+	06	24	-	-	0.0	0.0	-	-
	Pakpattan	02	04	+	+	0.0	0.0	-	-	0.0	0.0	-	-
	Sheikhupura	09	36	+	+	02	08	-	-	0.0	0.0	-	-
	Sialkot	01	04	+	+	02	08	-	-	0.0	0.0	-	-
	Sahiwal	01	04	+	+	0.0	0.0	-	-	0.0	0.0	-	-
	Lahore	10.0	03	+	+	12	05	+	+	05 (peafowl and pigeon)	20	+	+
	Multan	03	12	+	+	0.0	0.0	-	-	0.0	0.0	-	-
	Jhang	02	08	-	-	02	08	-	-	0.0	0.0	-	-

^1^birds kept under full human husbandry conditions and are fed with commercial food and supplements, ^2^Birds kept under partial human husbandry conditions and mainly reply on natural source of food. ^3^Wild birds that are kept under human control and provided commercial food and supplements. ^4^At least one of the studied flocks in particular district was positive, + and - indicate that samples were detected positive or negative by real-time PCR, respectively.

### Shipment of the samples and extraction of nucleic acid

A total 300 μL of whole blood was stored on QIACard FTA Indicator Four Spots (Qiagen, Hilden, Germany). These FTA Indicators have properties to inactivate the virus and preserve the nucleic acid. The samples were shipped at ambient temperature from Pakistan to the Department of Biomedical Sciences and Veterinary Public Health at the Swedish University of Agricultural Sciences (SLU) Uppsala, Sweden, for further processing and analysis.

The RNA was extracted from blood-impregnated FTA Indicator, as we have recently demonstrated [9]. Briefly, one punch (discs of 2.0 mm diameter) was taken from each sample using a Harris’ micropunch, according to manufacturer’s recommendation (BD09; Whatman). These discs were placed individually in a 1.5 mL microcentrifuge tube and the RNA was eluted with 52 μL of Tris–EDTA elution buffer (10 mMTris–HCl with pH 8.0, 0.1 mM EDTA, 50 units of RNase Inhibitor and 1 mM DTT) instead of company’s recommended RNA processing buffer. All reagents used here were purchased from Invitrogen, Carlsbad, CA, USA. These soaked discs were incubated for 15 min on ice and were flicked three times after every five minutes during the course of incubation). The extracted RNA was stored at –20°C until use for both sample screening and characterization using real-time PCR and sequencing, respectively.

### Screening of samples using real-time PCR

The detection of nucleic acid for NDV was performed using real-time PCR for M and F genes, as described previously [9,18]. The reaction was carried out in a Rotor-Gene 6000 real-time analyzer (Qiagen). The reporter dye (FAM) signals were measured at the annealing step of each cycle, and the threshold cycle (Ct) for each sample was calculated. The samples that had a Ct value <35 were considered positive in both M and F genes based real-time PCRs.

### Amplification and sequencing of F gene

All the samples that appeared positive for real-time PCR (both M and F gene bases) were further processed for the amplification of the complete F gene using degenerate primers as we described previously [19,20] with one modification. A total 7 μL of eluted RNA (the same as was used in real-time PCR screen) was added in a 25 μL reaction mix from One-Step RT-PCR Kit (Qiagen). However, the PCR conditions were kept same as reported previously [11,16,20]. The amplified PCR products were visualized in 1% (w/v) agarose gel in TBE-containing ethidium bromide. The bands of expected size were cut from the gel and were purified using the Wizard® SV Gel and PCR Clean-Up System (Promega, Co., Madison, WI, USA) according to the manufacturer’s instructions. The purified PCR products were sequenced with the same primers (used for PCR amplification) by the dideoxy-mediated chain-termination method using ABI PRISM BigDye® Terminator v3.1 Cycle Sequencing Kit (Applied Biosystems, Foster City, CA) as described by the manufacturer. Sequences were analyzed with an automated nucleic acid analyzer (ABI PRISM 3100; Applied Biosystems). To generate reliable consensus, each DNA fragment was sequenced at least twice in both the directions.

### Phylogenetic analysis

Sequence assembly and editing were performed using the SEQMAN program from DNASTAR Lasergene suite 9 (version 9.0.4 39; DNASTAR, Inc., Madison, WI, USA). To determine the phylogenetic relationships between APMV-1 viruses reported here and characterized previously from Asia and other parts of the world, a sequence stretch of the 373 bases in the F gene were retrieved from GenBank (http://www.ncbi.nlm.nih.gov). Sequences representing each known lineage were included in the analysis [7,21]. Known strains of NDV, representing each lineage/sub-lineage, were included in the trees to determine the distribution and clustering pattern of NDV strains studied in this manuscript. Furthermore, previously characterized stains of NDV were supported with accession numbers to facilitate further reading of a specific isolate. All sequences were aligned in BioEdit version 5.0.6 [22] using ClustalW and trimmed to equal length. A phylogenetic tree was then constructed using Bayesian Inference with the program MrBayes version 3.1.2 [23]. Two independent Monte Carlo Markov Chain (MCMC) chains were executed and sampled every 1000 generations using the default parameters of the priors’ panel. Once chains reached convergence (standard deviation values below 0.01), four million additional generations of the MCMC were run. Trees saved in this last step were used to construct a majority rule consensus tree. The analysis was based on the GTR + I + G model, which allow significantly changed posterior probability estimates. The nomenclature, based on lineages, was used in this study as described by Aldous *et al.* (2003). To further assess the genetic pattern in the tree, same sequences of the F genes were used for construction of phylogenetic tree using the neighbor-joining method (Kimura 2 parameter) with 2000 bootstrap replicates in MEGA4 software (CEMI, Tempe, AZ, USA) [24]. Finally, the labeling of the trees was made in FigTree v3.1.3.

### Determination of recombination events

Six methods (RDP, GeneConv, BootScan, MaxChi, Chimaera and SiScan) integrated in the RDP v3 program [25] were applied on the NDV sequences reported here to estimate any recombination event and to detect any putative recombination breakpoint. These methods were applied using following parameters: window size = 20, highest acceptable P-value = 0.001 and Bonferroni correction. For reliable results, any putative recombination events detected by more than one method were considered.

### Sequence submission

All the sequences (n = 23) used in this study were submitted to GenBank and are available under accession numbers from KC191601 to KC191623.

## Results

### Real-time PCR screening of clinical samples

Out of all samples analyzed, a total of 23 samples were found positive in both F and M gene based real-time PCRs. The results indicated that real-time PCR detected the samples from both commercial and rural poultry from all provinces. However, wild captive birds that were collected only from Punjab province were appeared positive. A detailed description of the collected, positive and negative samples is provided in Table 1. Majority of positive samples showed Ct values between 25 and 30. Results indicated that the FTA Indicators functioned as an appropriate sampling system for shipment at ambient temperature. All the samples with Ct values lower than 35 (threshold for positive samples) were used in conventional PCR for the amplification of F gene and subsequent sequencing.

### Phylogenetic relationship

**Figure 2:**
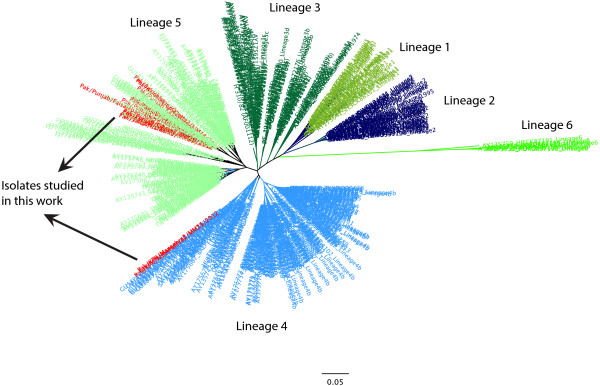
**A phylogenetic analysis of the partial sequence of F gene representing all the lineage of NDV.** The sequences reported in this study are colored red in both lineage 5 and lineage 4.

The Bayesian phylogenetic tree was constructed on 509 sequences, which were used by Aldous et al. (2003) and Cattoli et al. (2010) for the classification of NDV strains into six different lineages [7,21]. All previously characterized NDV isolates from Pakistan were also included in the phylogenetic analysis to ascertain the genetic diversity with the isolates characterized here and reported from rest of the world. The topology of the phylogenetic tree indicated the division of NDV strains clearly into six distinct lineages (Figure 2). Majority of the Pakistani NDV strains were grouped together and clustered within lineage 5, close to the previously characterized Pakistani isolates. Interestingly, the NDV isolates from Khyber Pakhtunkhwa province clustered with NDV strains of lineage 4.

**Figure 3:**
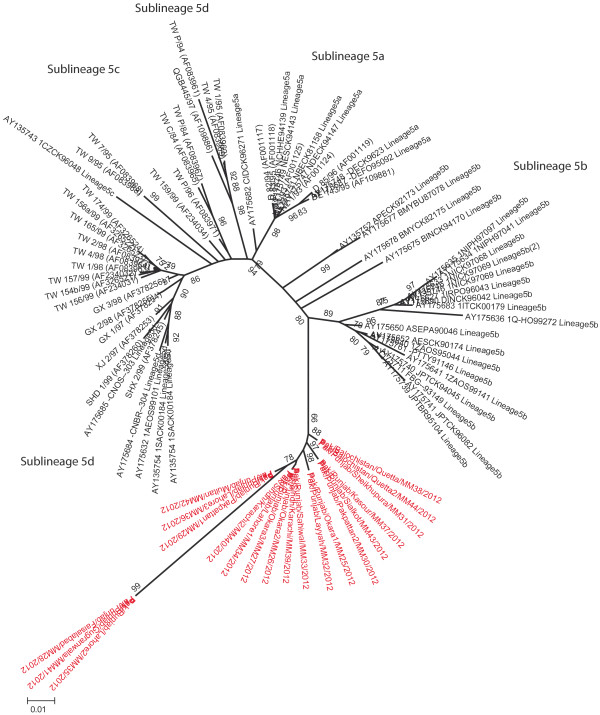
**A high-resolution phylogenetic tree for lineage 5.** Sequences presenting all the sub-lineages within lineage 5 are shown. Isolates shown in this study formed a separate cluster within sub-lineage 5b.

**Figure 4:**
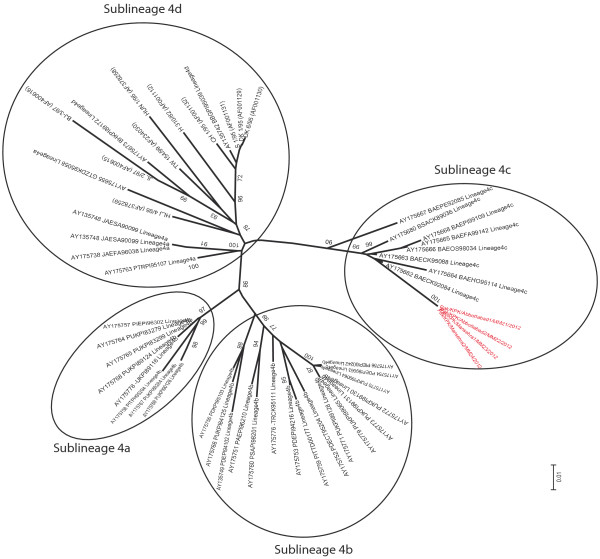
**A high-resolution phylogenetic tree for lineage 4.** Sequences presenting all the sub-lineages within lineage 4 are shown. Isolates shown in this study clustered within sub-lineage 4c.

Since both lineage 4 and 5 are further divided into different sub-lineages, a high-resolution phylogenetic analysis was conducted including the representative isolates of each sub-lineages for both lineage (4 and 5). Analysis indicated that all isolates, excepting isolates from Khyber Pakhtunkhwa province, clustered close to sub-lineage 5b. However, all these Pakistani isolates present a separate branch (Figure 3). The isolates from Khyber Pakhtunkhwa province (n = 4) were clustered with isolates belonging to sub-lineage 4c (Figure 4).

### Analysis of the amino-acid sequences of F protein

Proteolytic cleavage site motifs (amino acids 112–117) for the F0 protein of the all the isolates, putatively indicating the level of pathogenicity, were analyzed. Based on the cleavage site of the F protein, it was possible to demonstrate that all the isolates carry velogenic motif ^112^RRQKRF^117^ regardless of their phylogenetic distribution. However, three isolates collected from Faisalabad (MM26), Lahore (MM35), and Gujranwala (MM41) carry ^112^RKQKRF^117^motif at their cleavage sites. Both these motifs are generally identified in the strains of NDV that are highly virulent in chickens.

Comparison of the predicted amino acid sequences of the complete F gene showed that the seven neutralizing epitopes (D72, E74,A75, K78, A79, L343), believed to be critical for structure and function of F protein, were conserved and identical in all studied isolates. The potential N-glycosylation sites in F-glycoprotein (Asn-X-Ser/Thr or N-X-S/T, where X present any amino acid except aspartic acid or proline) were found at position85NRT87, 191NNT193, 366NTS368, 447NIS449, 471NNS473 and 541NNT543. Comparison of the complete F gene sequences among all the lineages showed that the studied strains of NDV shared highest nucleotide and predicted amino acid (95.7% and 91.3%) identity with the lineage5 whereas lowest (86.5% and 90.9%) with lineage 1 except for four isolates belonging to lineage 4.

### Recombination among F genes of NDV isolates

Evidence of recombination among poultry and ostrich NDV has been reported [26-28]. Recombination analyses performed on the F genes of the NDV isolates belonging to all lineages reported before and isolates characterized here appear to lack any recombination events.

## Discussion

Geographically, Pakistan is located (33°40′N and 73°10′E) at the crossroads of the strategically important regions of South, Central and Western Asia (Figure 1). From 2009 to mid 2012, a number of outbreaks of Newcastle disease have been reported to World Organization for Animal Health (OIE-WAHID interface, available at: http://www.oie.int/wahis/public.php) from Pakistan as well as neighboring countries. Most of the outbreaks have been reported from Iran and India that shares border with Pakistan. Despite some of the reports from selective regions only and emergence of novel NDV (5i) from Pakistan [9], it is of the essence to screen and characterize the NDV throughout the country.

The F gene sequence data of all the analyzed samples clustered virulent NDVs among the lineage 5 (sub-lineage 5b) except KPK province, where the ND strains were clustered together in lineage 4 (sub-lineage 4c). Throughout the world, the NDVs belonging to lineage 5 are considered to be the one involved in outbreaks in Far East [29-31] South Africa [32] and Europe [8]. Among many Asian countries and particularly those that shares border with Pakistan, there have been found and characterized number of velogenic NDVs within class II that belongs to various lineage and sub-lineages [7,33-35]; however, the dominant one is found to be lineage 5. Likewise, NDVs belonging to various sub-lineage like 5a, 5b, and novel 5i has been reported from Pakistan from different type of birds recently [9,11,16]. Contrary to recent study analysis of genetic nature of circulating NDVs except those of KPK province, where sub-lineage 5b was found circulating among rural, commercial and wild birds, we have seen a clear distinction between the viruses isolated from various types of birds, wild and commercial in the recent past. The isolates from wild birds were clustered within sub-lineage 5a and closely related to Indonesian isolate (AY562985) [15,17] while the ones from commercial and rural poultry were found within sub-lineage 5b, novel 5i and closely related to Swedish (GU585905) and Russian isolates (AY865652) [17,19,20].

Since 2005, lineage 4 (sub-lineage 4c) has been found now and from a different geographical region, Abbottabad and Mansehra districts of KPK province. Previously, the lineage 4 was isolated and characterized from areas in and around coastal border of Pakistan [14]. The re-emergence of lineage 4 (sub-lineage 4c) from KPK province might be attributed either to the fact that viruses have not been characterized from this particular region before or either to the movement of migratory/caged birds across the country or from north to south involving Europe, Asia and Middle East. Historically, lineage 4 has been isolated and characterized among domestic fowls, ostrich, falcons and pigeons from middle east countries (United Arab Emirates and Saudi Arabia), Asian (Japan and China) and European countries (UK, Italy, Peru and Belgium) [1,34]; however, most of NDVs belonging to sub-lineage 4c have been reported from wild captive and domestic fowls from United Arab Emirates [7]. Isolation and characterization of NDV (JP/Chiba-pa/97) from parakeets exported to Japan in 1997 [29] gives an evidence that lineage 4 is present in Pakistan since 1997. Since it is the only report from past, it could be hypothesized that lineage 4 is present even before and is still circulating in the environment. Further, it is imperative to describe the fact that samples were collected during a designated period of six month throughout the country and all of the clinical outbreaks are not necessarily included in the study. This means that there may exist more diversity in NDV strains in Pakistan and therefore, presence of lineage 4 (sub-lineage 4c) in already reported area (Karachi) or others cannot be ignored. The isolation and subsequent characterization of isolates from turkey and domestic fowls in UK (Q-GB506/97) [1], exported parakeets from Pakistan to Japan (JP/Chiba-pa/97) [29], and from pigeons in china (C/98-1) [34] which clustered together with Italian exotic isolate (IT-148/94) [8] in lineage 4 provides a possible epidemiological transmission through migratory birds from north to south. Considering migratory/caged birds as reservoir/carrier of NDVs in an inapparent form and the potential of viruses to infect multiple avian species without prior adaptation [1,2,26,36], raises the concerns in worldwide distribution of velogenic pathotypes through trading and migration of birds across regional and international boundaries.

Recent outbreaks of ND in Pakistan are supposed to be due to a breach in the biosecurity measures. However, the role of vaccines in providing protection against the field challenge also needs to be evaluated. Inability of live vaccines to elicit protective immune response might be due to several reasons like improper cold chain supply system, inappropriate route of vaccination, or uneven vaccination schedules. Administration of both live and inactivated ND vaccine could be practiced to protect the birds from virulent NDVs [37]. Presently, lentogenic (LaSota) or mesogenic strains (Muktesewar) of NDVs are being used to vaccinate the birds in Pakistan. However, it is still a matter of question whether these vaccinal strains are able to elicit a protective immune response against the prevailing field strains with high genetic gap in relation to vaccinal strains as evidenced and reported previously [9]. It has been reported that the currently practiced NDV vaccines give better protection against the velogenic NDVs isolated in 1930 to 70s (Herts33/56, California 71) than the ones, which have been isolated in past few years [5,9]. Hence parameters for selection of a vaccinal strain are needed to be reconsidered. Furthermore, monitoring the immune response of birds to NDV vaccines along with strict biosecurity measures should be employed to keep the flocks free of vNDVs infection.

## Conclusions

Simultaneous detection of multiple velogenic strains of NDV (lineage 4 and 5) from various regions of the country warrants continuous isolation and molecular epidemiological investigations involving wild/caged birds. Vaccine strategies that can elicit high antibody response along with continuous monitoring from the laboratory coupled with improved biosecurity measures in the form is suggested.

## Abbreviations

NDV: Newcastle disease virus; F: Fusion protein; M: Matrix protein; APMV-1: Avian paramyxoviruses serotype 1; KPK: Khyber Pakhtunkhwa.

## Competing interests

The authors declare that they have no competing interests.

## Authors’ contributions

MZS, TY, JN, MABS, MS, MS, MA, MA, MTK, AAA, AG, AA, AAC, AAA, MZK, AA, MUG, ZI, HA, KZ, NM, SAK, NA, WY and MUS collected samples from various geographical areas of Pakistan. MZS, MM wrote the manuscript. MB, SZ discussed and reviewed the manuscript. MM and MZS designed the manuscript and analyzed the data. All authors read and approved the final manuscript.
